# Closing the Gap: Multiresonant TADF Emitters and TADF Dendrimers in Solution-Processed OLEDs

**DOI:** 10.1021/accountsmr.6c00190

**Published:** 2026-09-25

**Authors:** Mahni Fatahi, Eli Zysman-Colman

**Affiliations:** Organic Semiconductor Centre, EaStCHEM School of Chemistry, 7486 University of St Andrews, St Andrews, KY16 9ST, UK

## Abstract

Organic light-emitting diodes (OLEDs) have become a cornerstone technology in modern displays, yet their dominant manufacturing route, vacuum deposition (VD), remains cost-intensive and intrinsically wasteful. Solution processing (SP) offers an attractive alternative because it enables high-throughput fabrication, reduced material loss, and compatibility with large-area and flexible substrates. Organic thermally activated delayed fluorescence (TADF) emitters are uniquely positioned for SP-OLEDs, as they can harvest both singlet and triplet excitons, providing a route to near-unity device internal quantum efficiency, without the need for scarce heavy metals. The key challenge, however, is that the performance of SP TADF OLEDs still lags behind VD devices in both maximum external quantum efficiency and efficiency roll-off. These performance gaps originate from SP-specific constraints such as unoptimized film morphology, aggregation-caused quenching (ACQ), limited control over transition dipole orientation, and charge imbalance in multilayer stacks. Consequently, closing the VD–SP performance divide requires coordinated progress in molecular engineering and device engineering.

This Account summarizes recent materials-design strategies for high-performance SP TADF OLEDs, with emphasis on multiresonant TADF (MR-TADF) emitters and TADF dendrimers. Small-molecule donor–acceptor (D-A) TADF emitters, though compatible for SP-OLEDs, often show broad emission resulting from their emissive long-range charge-transfer (LRCT) states and large reorganization energies, compromising color purity. MR-TADF emitters address this limitation, providing narrowband emission from a short-range charge-transfer (SRCT) state resulting from the alternating HOMO/LUMO density on adjacent atoms. Yet even state-of-the-art MR-TADF materials that deliver outstanding VD-OLED performance yield SP-OLEDs showing a marked drop in performance, evidencing that processing-induced disorder and bimolecular quenching become dominant in these devices. We therefore highlight how contemporary MR-TADF emitter design has shifted beyond solubility improvements toward deliberate control of film-state organization (e.g., liquid-crystalline self-assembly to induce preferential horizontal dipole orientation and improve outcoupling) and excited-state kinetics (e.g., multichromophore coupling and intramolecular FRET concepts to accelerate effective triplet harvesting and reduce roll-off while retaining narrow emission).

In parallel, TADF dendrimers combine the high solubility and film-forming ability of polymers with the high purities possible with small molecules. This leads to SP devices showing comparable performance to VD ones. We discuss how dendrimer design has progressed from early low-performing prototype devices to SP-OLEDs showing *EQE*_max_ approaching 30%. We discuss how dendrimer design provides additional levers to modulate charge balance and efficiency roll-off. Importantly, dendrimers have also proven to be versatile beyond OLEDs, for example, in scalable bar-coated light-emitting electrochemical cells and in sensing, illustrating their broader relevance within organic electronics. Finally, we show that MR-TADF dendrimers, particularly when combined with hyperfluorescence (HF) device concepts, can integrate narrowband emission, high photoluminescence efficiency, aggregation suppression, and improved exciton management, enabling SP-OLED performances approaching VD benchmarks.

Many bottlenecks of SP-OLEDs, ACQ, limited orientation control, and slow MR-TADF RISC have now been addressed individually. The next step is to integrate these insights into unified emitter host device design rules. MR-TADF emitters and dendritic architectures, especially in HF concepts, provide a promising pathway toward truly scalable, high color-purity OLED manufacturing.

## Graphical abstract

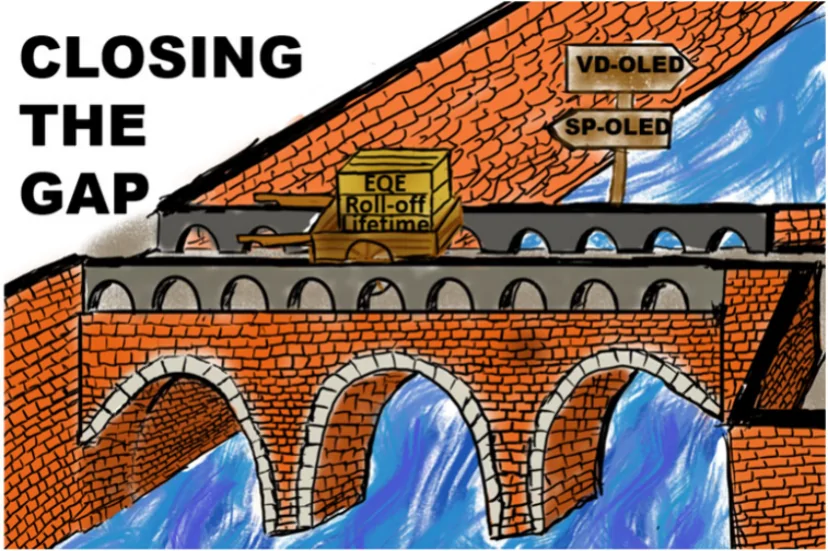

## Introduction

Organic thermally activated delayed fluorescence (TADF) emitters have emerged as one of the most promising classes of materials as emitters in organic light-emitting diodes (OLEDs).^1^ Their significance arises primarily from their intrinsic ability to harvest singlet and triplet excitons through intersystem crossing and reverse intersystem crossing (RISC) cycling, enabling devices with internal quantum efficiencies approaching 100%.^1^ Unlike organometallic phosphorescent emitters that contain scarce platinoid metals, organic TADF materials are mainly composed of abundant p-block elements, and are more sustainable.^1^

Organic TADF emitters show exceptional structural diversity, which allows for precise engineering of chemical, physical and optoelectronic properties, including examples that emit across the full visible spectrum^1,2^ including those that meet the color purity and efficiency requirements of modern display technologies.^3–5^ As a result, vacuum-deposited (VD) OLEDs employing TADF emitters have reached performance levels competitive with phosphorescent devices.^1^

Despite these advances, vacuum thermal evaporation is a cost-intensive fabrication technique associated with significant materials waste, high energy consumption, and limited scalability of the panel size.^6^ Consequently, efforts to lower the manufacturing costs not to mention delivering displays compatible with new application formats remain key challenges for the OLED sector. In this context, solution-processing (SP) as manufacturing technique, including spin coating, blade coating, and inkjet printing, offers compelling advantages for organic electronic devices, including OLEDs. This includes less materials waste, lower processing costs, higher production throughput, and compatibility with the manufacture of large-area and flexible displays.^7^ However, the performance of SP TADF OLEDs still lags behind their VD TADF OLED counterparts across several critical device metrics, including maximum external quantum efficiency *EQE*_max_, efficiency roll-off, and operational stability. These limitations originate from a combination of factors, such as suboptimal solid-state packing, aggregation-caused quenching (ACQ), and suboptimal charge balance.^8^ Addressing these challenges requires concerted efforts spanning both materials design and device engineering.

This Account primarily focuses on advances in SP TADF materials design, an area that has seen significant progress in recent years. We discuss molecular strategies developed to overcome the intrinsic limitations of SP TADF emitters and to enable the fabrication of high-performance SP TADF OLEDs. In this context, we broadly categorize the TADF materials as either small-molecule emitters, polymers, or dendrimers. Although conjugated polymers played a pioneering role in the development of SP organic semiconductors in the early 1990s,^9,10^ they often suffer from inherent drawbacks that limit their suitability for high-performance and industrially reproducible OLEDs.^11,12^ Polymerizations typically lead to ill-defined molecular weights and compositions, resulting in broadened or undesirably color shifting of the emission spectra, and batch-to-batch variability, which ultimately leads to inconsistent Commission Internationale de l’Éclairage (CIE) color coordinates, a parameter crucial to modern displays. In addition, residual catalysts and side products can be difficult to remove completely during polymer synthesis, and may introduce trap states that adversely affect device performance and stability.^12^ For these reasons, we focus only on small-molecule and dendrimer TADF emitters. The latter materials class combines the structural precision and reproducibility of small molecules with enhanced processability and morphological control endemic to macromolecules, offering a promising route toward bridging the performance gap between SP and VD TADF OLEDs. Particular emphasis is placed on multiresonant TADF (MR-TADF) emitters, which due to their intrinsic bright, narrowband emission, represent one of the most active and impactful material platforms currently under investigation for high-efficiency SP-OLEDs. A key strategy for achieving high-efficiency OLEDs is the use of sensitization strategies (so-called hyperfluorescent (HF) or hyperphosphorescent (HP) devices), which in this account refer to devices containing within the emissive layer an additional TADF emitter that is responsible for exciton harvesting and then transferring them to the terminal emitter via Förster Resonance Energy Transfer. In so doing, the desired emission properties of the terminal emitter are preserved in the HF device.

## Small-Molecule MR-TADF Emitters

Many of the early examples of TADF emitters are based on strongly twisted donor–acceptor architectures where donor and acceptor fragments, like carbazole and triazine, respectively, are planar and rigid, which leads to low solubility in organic solvents, negatively impacting film formation. The introduction of solubilizing substituents is frequently required to make these emitters compatible with SP techniques e.g. decoration with *tert*-butyl^13^ or adamantyl groups,^14^ which have been shown to increase solubility due to their ability to disrupt crystal packing. Alternatively, attaching long alkyl chains, including those used in liquid-crystalline TADF emitters, has been employed to enhance solubility in organic solvents.^15–19^ However, due to their large reorganization energies, most donor–acceptor TADF emitters have broad photoluminescence characterized by large full widths at half maxima (FHWM), which adversely affects color purity and thus their suitability in high-definition displays. Additionally, the structural diversity of this class of emitters is limited as twisted conformations, promoting spatial separation of the HOMO and LUMO electron densities are required to render singlet-triplet energy gaps (Δ*E*_ST_), suitably small to promote TADF.^20^

To overcome the broadband emission endemic to most D-A TADF emitters, Hatakeyama and co-workers first introduced in 2015 a novel emitter design, referred to as MR-TADF.^21^ MR-TADF emitters are p- and n- doped polycyclic aromatic hydrocarbons where donor and acceptor motifs are positioned either *meta* or *para* with respect to each other, leading to an alternating pattern of electron density on neighbouring atoms when comparing the HOMO and LUMO. This results in the S_1_ state adopting a SRCT character, distinct to the LRCT character of D-A TADF emitters. The typically rigid structure leads to narrowband emission as a result of a small reorganization energies. In these molecules, the p-type dopants are mainly nitrogen, oxygen, or sulfur atoms, while the n-type dopants are boron atoms or carbonyl groups. However, these rigid and planar cores generally show poor solubility in organic solvents, which effectively excludes their use in SP-OLEDs. It is thus necessary to introduce solubilizing groups about these cores for SP-OLEDs to be fabricated. In recent years, the design and use of MR-TADF emitters have gained significant attention within the SP-OLED landscape. Their narrowband emission, high Φ_PL_ and good color tunability makes them ideal candidates for modern day OLEDs and significant progress has been made to lower the efficiency and roll-off gap between SP devices and their VD counterparts.

Several reports on MR-TADF OLEDs clearly illustrate that an outstanding performance of VD-OLEDs does not necessarily translate to SP-OLEDs using the same emitter. For instance, Jin *et al*. reported deep-blue **DOBN** ^22^ (Figure 1) based on a hybrid LRCT/SRCT emissive excited state design that delivered HF VD-OLEDs achieving an *EQE*_max_ of 35.4% at blue CIE coordinates of (0.15, 0.04) using the former as the terminal emitter. The SP HF OLEDs (using a structurally-related sensitizer) reached an *EQE*_max_ of 27.7%; however, a distinct performance gap persisted, particularly with respect to maximum luminance (VD-OLED: *L*_max_ = 1231 cd m^–2^ *vs.* SP-OLED: 591 cd m^–2^). The corresponding binary devices provide an even more drastic picture, where the VD devices achieve an *EQE*_max_ of 24.1%, whereas the *EQE*_max_ of the SP devices did not even reach 7%.

**Figure 1:**
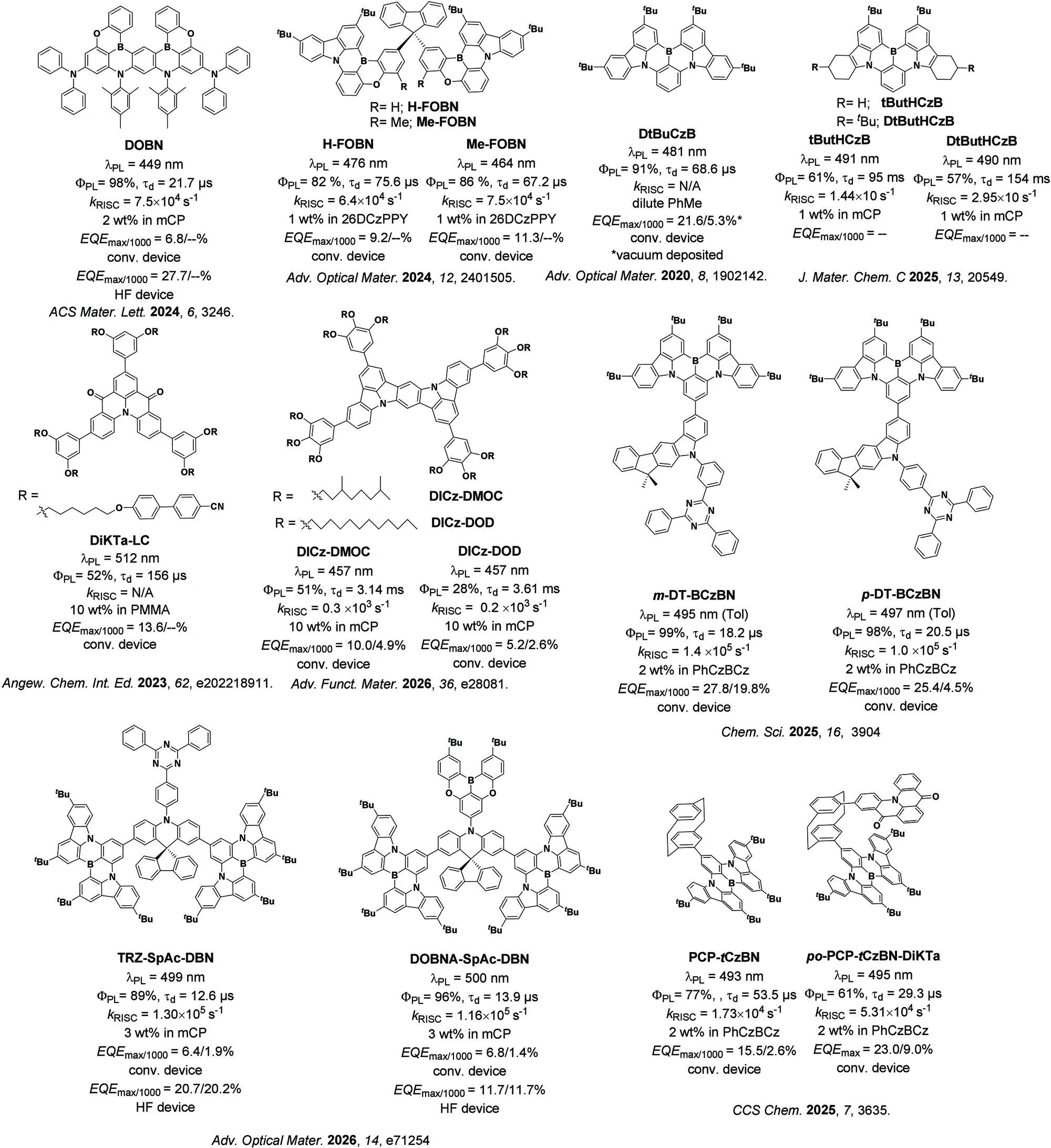
Chemical structures, key photophysical and device data of MR-TADF emitters for SP-OLEDs.^19,22–29^

A similar disparity is observed in the dual-MR-TADF core, **Me-FOBN**,^23^ by Song *et al.*, where suppressed aggregation translates into a high-efficiency (*EQE*_max_ of 25.1%) blue VD device (Figure 1). However, the corresponding SP-OLEDs exhibited substantially lower *EQE*_max_ of 11.3%, despite maintaining comparable emission color [CIE = (0.14, 0.08), VD *vs*. (0.14, 0.09), SP] and spectral width (FWHM = 28 nm, VD *vs*. 32 nm, SP). Importantly, the efficiency roll-off in the SP devices was markedly stronger (*L*_max_ = 3560 cd m^–2^, VD *vs*. 312 cd m^–2^, SP), underscoring the impact of poorer film morphology and increased quenching probability in the latter. These two examples demonstrate that increasing solubility and suppressing aggregation to improve film morphology, while necessary, are not sufficient to bridge the performance gap between VD and SP MR-TADF OLEDs.

Consequently, closing this gap requires advances in emitter design, these must be designed not only for narrowband emission and efficient triplet harvesting, but also for controlled organization in film and reduced sensitivity to processing-induced disorder. In parallel, device engineering strategies, such as host design and selection optimized for SP devices and refined multilayer SP fabrication protocols, are essential to mitigate charge imbalance and exciton quenching in SP device stacks. Only the right combination of optimized molecular architecture and device structure can realize the full potential of SP MR-TADF OLEDs

In a recent study, we introduced partially saturated carbazole derivatives as donors to the widely studied **DtBuCzB**,^28^ to blue-shift the emission and improve the solubility.^24^ **DtButHCzB** and **tButHCzB** (Figure 1), show λ_PL_ of 480 and 479 nm (FWHM = 21 nm for both) and high Φ_PL_ of 87 and 91% in toluene, respectively. However, this structural modification also resulted in large Δ*E*_ST_ of 310 and 320 meV, respectively, leading to slower *k*_RISC_ on the order of 10^1^ s^–1^ in 1 wt % doped films in mCP. Additionally, pronounced aggregation was observed, causing a red-shifted and broadened emission (λ_PL_= 490 and 491 nm, FWHM of 34 and 45 nm, respectively) and a substantial drop in Φ_PL_ to 57 and 61%, respectively. Strong π–π stacking, present even at this low doping concentration, indicates that partial saturation alone is insufficient to suppress ACQ, rendering these emitters unsuitable for high-performance SP-OLEDs.

In 2023, our group reported a notable strategy to overcome a long-standing bottleneck of SP small-molecule OLEDs, namely that spin-coated films typically yield isotropically oriented emitters, which means that their transition dipole moments (TDM), are likewise randomly oriented and thus there is no enhanced light-outcoupling in the devices.^30,31^ By decorating the MR-TADF emitter, **DiKTa**, with cyanobiphenyl-based mesogenic units, we reported the first example of a liquid-crystalline MR-TADF emitter **DiKTa-LC** (Figure 1).^25^ Importantly, the mesogenic substituents are electronically decoupled from the MR-TADF core, preserving the characteristic SRCT excited state and narrowband emission (FWHM = 33 nm in toluene). Structural and thermal analyses confirmed the formation of a nematic mesophase. As a direct consequence of this liquid-crystalline ordering, spin-coated neat films of **DiKTa-LC** exhibit a pronounced horizontal orientation of the TDM (anisotropy factor *a* ≈ 0.28, Figure 2). This preferential orientation is retained in 10 wt % doped films in PVK and leads to enhanced light out-coupling without altering the emissive layer architecture. SP-OLEDs fabricated with **DiKTa-LC** emitted at CIE coordinates of (0.22, 0.49) and achieved an *EQE*_max_ of 13.6%, indicating an outcoupling efficiency of over 31%, increased from 20-25% for devices with isotropically oriented emitters.

**Figure 2:**
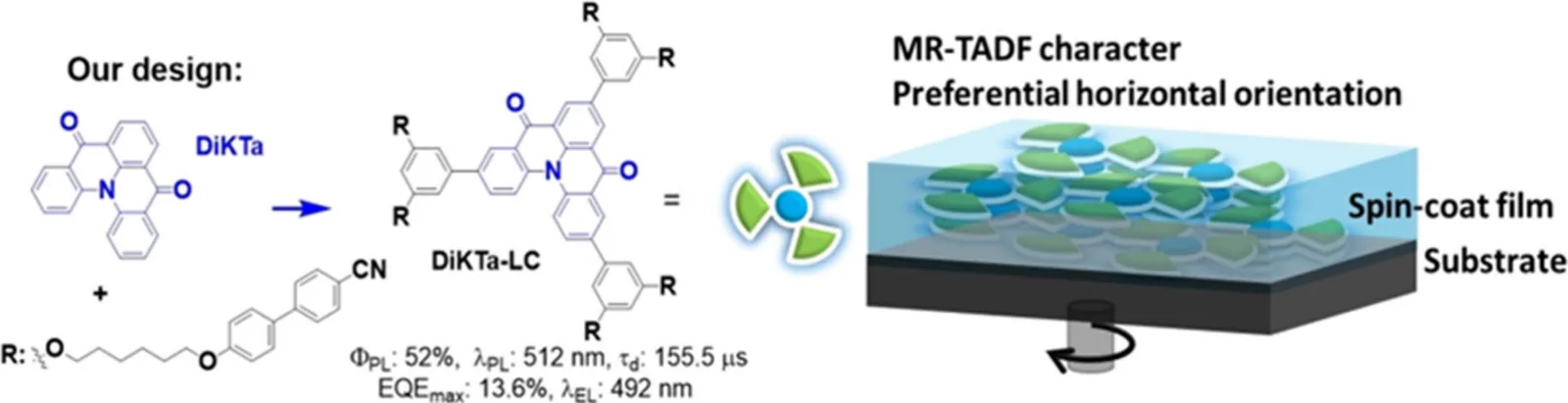
Design principle and schematic drawing of preferential horizontal orientation of **DiKTa-LC** in spin-coated films. Adapted from ref.^25^ Available under a CC-BY 4.0 license. Copyright 2023 D. Chen *et al*.

Recently we reported a related study, where the blue diindolocarbazole MR-TADF core was decorated with mesogenic units to form discotic liquid crystals.^19^ **DICz-DMOC** (Figure 1) forms a room-temperature columnar mesophase that self-assembles in a homeotropic fashion, resulting in a preferential horizontal alignment of the transition dipole moments (*a* = 0.22, corresponding to 78% horizontal orientation). This supramolecular ordering translates into enhanced light outcoupling and an improved SP device performance. SP-OLEDs with 10 wt % **DICz-DMOC** in mCP achieved an *EQE*_max_ of 10.0% at CIE coordinates of (0.15, 0.19), while neat-film devices reached an *EQE*_max_ of 5.3%, outperforming the structurally related soft-crystalline analogue **DICz-DOD** (Figure 1).

Together these results show that molecular self-assembly can control MR-TADF emitter orientation in SP films, leading to improved SP-OLED efficiencies, underscoring the potential of MR-TADF liquid crystals as an orientation-directing platform.

While orientation control addresses optical light out-coupling losses, another central issue with MR-TADF emitters is their intrinsically slow RISC, which originates from weak spin-orbit coupling between the singlet and triplet states and moderate Δ*E*_ST_ values,^33^ and leads to pronounced efficiency roll-off in the device,^34^ particularly in SP-OLEDs. In this context, we introduced a fundamentally different molecular design strategy by bridging two MR-TADF cores within a single molecule, *po* **-PCP**-*t* **CzBN-DiKTa** (Figure 1),^27^ as a strategy to accelerate RISC. By electronically coupling two MR TADF units (**DtBuCzB** and **DiKTa**) through a rigid paracyclophane linker, the density of intermediate excited states increased, while the SRCT character of the **DtBuCzB** moiety is retained (Figure 3a). This design leads to a significantly enhanced spin-vibronic coupling between singlet and triplet manifolds, resulting in faster RISC (*k*_RISC_ of 5.31 × 10^4^ s^–1^) compared to monomeric MR-TADF reference compound (**PCP**-*t* **CzBN**, *k*_RISC_ = 1.73 × 10^4^ s^–1^). Photophysical investigations revealed shortened delayed fluorescence lifetimes of 29.3 μs for *po* **-PCP**-*t* **CzBN-DiKTa** (**PCP**-*t* **CzBN**: τ_d_ = 53.5 μs) and narrowband λ_PL_ of 495 nm (FWHM of 28 nm) in 2 wt % doped films in PhCzBCz, indicating more effective triplet harvesting without sacrificing color purity. When implemented in SP-OLEDs, the device with *po* **-PCP**-*t* **CzBN-DiKTa** delivered higher *EQE*_max_ of 23.0% compared to 15.5% for the device with **PCP**-*t* **CzBN** (same device stack) and reduced efficiency roll-off (*EQE*_1000_ = 9.0% *po* **-PCP**-*t* **CzBN-DiKTa**; 2.6% **PCP**-*t* **CzBN**), highlighting that engineering the excited-state manifold through multichromophore coupling is an effective strategy to overcome intrinsic kinetic limitations of MR-TADF systems.

**Figure 3:**
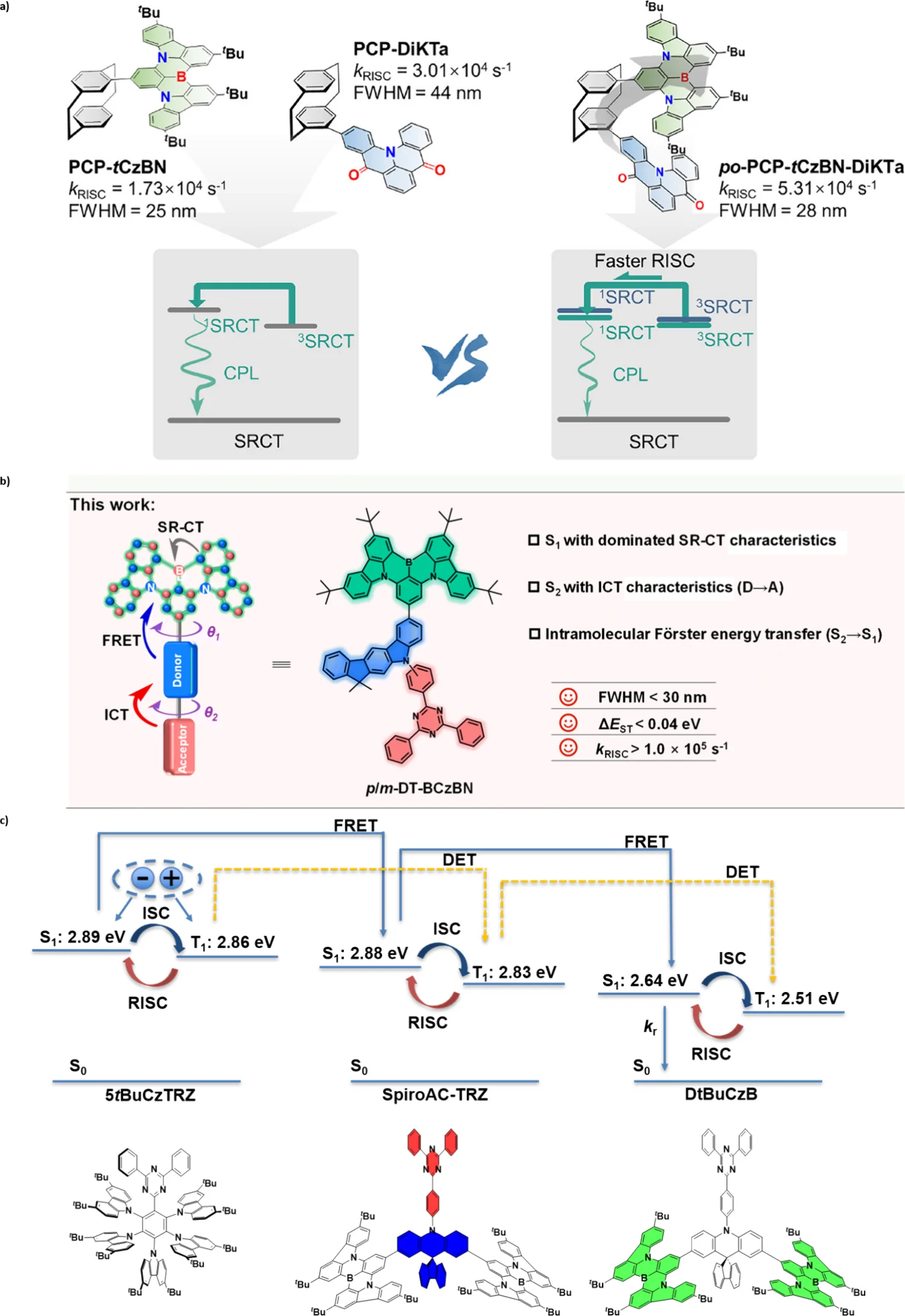
a) Schematic excited state levels of **PCP-DiKTa**, **PCP**-*t* **CzBN**, and *po* **-PCP**-*t* **CzBN-DiKTa** and introduction of close by higher lying excited states to accelerate RISC. Reproduced from Ref.^32^ Available under a CC-BY-NC 3.0 license. Copyright 2025 Y. Xu *et al*. b) The proposed molecular design concept of MR-TADF skeleton-donor–acceptor moieties for high-efficiency narrowband MR-TADF emitters (*p* **/** *m* **-DT-BCzBN**). Adapted from Ref.^26^ Available under a CC-BY-NC 3.0 license. Copyright 2025 Z. Yang *et al.* c) Proposed energy cascade between the sensitizer, **5** *t* **BuCzTRZ**, the central D-A TADF motive, **SpiroAC-TRZ**, and the MR-TADF motive **DtBuCzB**. c) Proposed energy cascade between the sensitizer, **5** *t* **BuCzTRZ**, the central D-A TADF motive, **SpiroAC-TRZ**, and the MR-TADF motive **DtBuCzB**. Adapted from Ref.^29^ Available under a CC-BY 4.0 license. Copyright 2026 M. Fatahi *et al*.

Yang *et al*.^26^ addressed the challenge of slow RISC kinetics by introducing an intramolecular charge-transfer (ICT) assisted MR-TADF design, in which a donor–acceptor moiety is grafted onto a **DtBuCzB** core. In this architecture, the emissive S_1_ state retains dominant SRCT character, preserving the narrowband emission (FWHM = 21-25 nm in toluene). A higher-lying ICT state provides an efficient spin-flip channel for triplet excitons resulting in much faster *k*_RISC_ values of 1.4 × 10^5^ and 1.0 × 10^5^ s^–1^ for *m* **-DT-BCzBN** and *p* **-DT-BCzBN** (Figure 1), respectively compared to 1.4 × 10^4^ s^–1^ for the parent **BCzBN** (*aka*. **DtBuCzB**). Sensitizer-free SP-OLEDs based on these emitters reached *EQE*_max_ of 27.8% (*m* **-DT-BCzBN**) and 25.4% (*p* **-DT-BCzBN**), providing narrowband electroluminescence at 492-494 nm (FWHM = 29 nm) and substantially suppressed efficiency roll-off (*EQE*_500_ = 21.5% *m* **-DT-BCzBN**); 11.0% *p* **-DT-BCzBN**), demonstrating that carefully positioned ICT states can accelerate RISC without compromising color purity (Figure 3b).

Complementary to this approach, we reported an intramolecular Förster resonance energy transfer (FRET) cascade emitter design to manage exciton kinetics in SP MR-TADF emitters. Here, similar to the aforementioned example from Yang *et al.*,^26^ a fast RISC donor–acceptor TADF unit is covalently linked to two peripheral MR-TADF emitters, ensuring efficient intramolecular energy transfer while maintaining narrowband emission from **DtBuCzB** (Figure 3c). The two emitters, **TRZ-SpAc-DBN** and **DOBNA-SpAc-DBN** (Figure 1),^29^ display narrowband emission with λ_PL_ of 489 nm and FWHM of 24 nm in toluene. In 3 wt % doped films in mCP the emitters have high Φ_PL_ of 89 and 96%, and *k*_RISC_ values of 1.3 and 1.02 × 10^5^ s^–1^, respectively. When employed as terminal emitter in HF SP-OLEDs using **5** *t* **BuCzTRZ** as a sensitizer, the device with **TRZ-SpAc-DBN** showed an *EQE*_max_ of 20.7% and an exceptionally low efficiency roll-off of less than 2.5% at 1000 cd m^–2^, resulting in an *EQE*_1000_ of 20.2%; notably, the *EQE* remained at 13.6% at a very high luminance of 10,000 cd m^–2^.

Together, these studies highlight that incorporating low-lying LRCT states by incorporating D-A TADF moieties into the emitter design represent a powerful, complementary strategy to decouple the narrowband emission emanating from the MR-TADF fragment from the triplet-harvesting kinetics managed by the D-A TADF unit(s), providing an attractive pathway to overcome the intrinsically limited RISC of MR-TADF emitters that affect SP-OLED performance.

In summary, the presented selection of studies (summarized in Table **S1**) illustrates that recent advances in small-molecule MR-TADF emitters for SP-OLEDs have progressed well beyond early strategies focused primarily on improving solubility. State-of-the-art molecular design concepts now address film-state organization and excited-state kinetics as interdependent factors that affect device performance. Approaches based on liquid-crystalline emitters allow control over the transition dipole orientation and light out-coupling, while multichromophore coupling, and intramolecular FRET designs provide effective routes to accelerate RISC without compromising the narrowband emission of MR-TADF cores. Collectively, these strategies have substantially narrowed the efficiency and roll-off performance gaps between SP and VD MR-TADF OLEDs. Nevertheless, a persistent limitation of small-molecule MR-TADF emitters remains their pronounced tendency toward aggregation in SP films, which can induce spectral broadening and non-radiative deactivation of excited states. Additionally, comparative studies highlight that even for high-performance emitters, the device architecture and processing technology used to fabricate SP-OLEDs introduce loss channels that are largely absent in VD-OLEDs. Addressing these challenges will be essential for translating the promising photophysical properties of MR-TADF emitters into high-performance SP-OLED technologies.

## TADF Dendrimers

Dendrimers claim their name from their dendritic, branch-like structure. Similar to polymers, dendrimers are categorized as macromolecules and thus possess high solubilities in organic solvents; however, in contrast to polymers, dendrimers have well-defined molecule structure and composition, meaning they can be purified in a similar manner as small molecules and, therefore, have well-defined and described photophysical properties. This makes dendrimeric materials ideal for the use in SP devices. TADF dendrimers were firstly introduced by Albrecht *et al*.^35^ in 2015 who reported a set of dendrimers consisting of a triphenyl triazine acceptor, which is decorated with carbazole donor dendrons of different generations, **G2TAZ**, **G3TAZ**, and **G4TAZ** (Figure 4). These first examples of TADF dendrimers translated into SP devices showing poor performances with *EQE*_*max*_ of less than 3.5%. The concept of dendrimerization, however, has gained considerable traction in the field of TADF emitters, as emitter design has iterated and device performance has steadily improved, with over 50 reports over the recent 10 years.^1,36^

**Figure 4:**
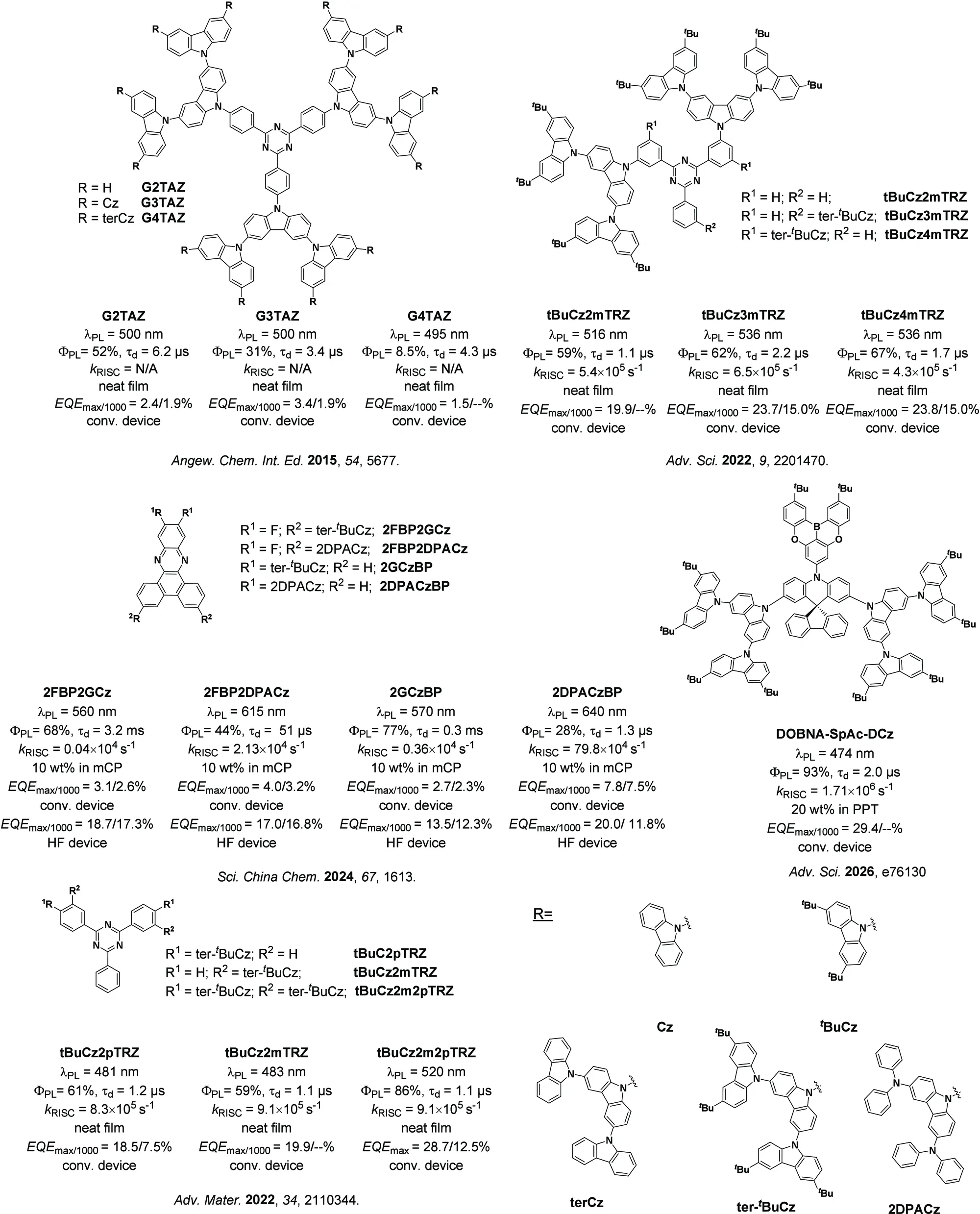
Chemical structures, key photophysical and device data of some D-A TADF dendrimers for SP-OLEDs, including those from our group.^35,37–40^

Building upon these early concepts, subsequent work by our group demonstrated that the initially low device performance with these first TADF dendrimers was in part a function of inefficient TADF in these particular dendrimer architectures. Informed by a detailed photophysical and device study on *meta*- versus *para*-disposed donor dendrons in donor–acceptor TADF dendrimers,^37^ a decisive breakthrough was achieved by combining *meta*- and *para*-connected second generation carbazole donor dendrons within a single molecule. **tBuCz2m2pTRZ** ^38^ (Figure 4) shows green emission at λ_PL_ of 520 nm and a very small Δ*E*_ST_ of 40 meV in neat film to enable fast TADF. The efficient TADF results from a synergy between a small Δ*E*_ST_, high SOC, and small reorganization energies due to the incorporation of the *meta*-decorated donor dendrons, while the *para*-decorated donor-dendrons confer high oscillator strength, leading to very bright emission. The SP-OLEDs showed green λ_EL_ of 540 nm [CIE coordinates of (0.37, 0.57)] and an *EQE*_max_ of 28.7%. Notably, after modest charge-balance optimization by adding OXD-7 as a co-dopant in the emissive layer, the efficiency roll-off improved and the *EQE*_500_ remained high at 22.7%, clearly demonstrating that the use of dendrimer emitters can rival state-of-the-art small-molecule systems in SP-OLEDs.

Subsequently, we demonstrated longer emission wavelength TADF dendrimers, addressing a long-standing challenge for red SP-OLEDs.^39^ We reported a series of orange-to-red TADF dendrimers based on a dibenzo­[*a,c*]­phenazine acceptor, where systematic variation of donor strength and substitution pattern led to pronounced differences in RISC kinetics. Dendrimers with donors substituted at the 11,12 positions have *k*_RISC_ that are more than an order of magnitude faster than those substituted at the 3,6 positions. The device with the best-performing emitter, **2DPACzBP** (Figure 4), showed red electroluminescence at 640 nm [CIE coordinates of (0.61, 0.36)] and an *EQE*_max_ of 7.8%, which was effectively maintained at 1000 cd m^–2^ (*EQE*_1000_ = 7.5%). When used as a terminal emitter in a HF device using **4CzIPN** as an assistant dopant, the ternary device delivered an *EQE*_max_ of 20.0% at a λ_EL_ of 605 nm [CIE coordinates of (0.53, 0.45)], placing these devices among the most efficient orange-to-red SP-OLEDs reported to date.^13^

Recently we reported a blue TADF dendrimer, **DOBNA-SpAc-DCz** (Figure 4),^40^ which has a high Φ_PL_ of 93% and a short τ_d_ of 2.04 μs in 20 wt % doped films in PPT. The resulting fast *k*_RISC_ of 1.7 × 10^6^ s^–1^ enabled the fabrication of very efficient SP-OLEDs, showing an *EQE*_max_ of 29.4% at CIE coordinates of (0.14, 0.21). Clearly demonstrating the potential of TADF dendrimer SP-OLEDs.

Taken together, these results (summarized in Table **S2**) illustrate how donor–acceptor TADF dendrimers have evolved from early molecular designs that produced devices showing low *EQE*_max_ values into a class of emitters capable of delivering SP devices with *EQE*s approaching 30%. The combination of defined molecular structure, excellent film-forming properties, and tunable excited-state kinetics makes TADF dendrimers an attractive class of emitters for use in for high-performance SP-OLEDs.

Beyond their successful implementation in SP-OLEDs, TADF dendrimers have increasingly demonstrated versatility across a broader range of optoelectronic and functional applications, underscoring the intrinsic advantages of dendrimeric structures. One example is our recent collaborative work about using **tBuCz2m2pTRZ** (Figure 4) as the emitter in light-emitting electrochemical cells (LECs) fabricated via scalable bar-coating.^41^ In contrast to conjugated polymers or small molecules, the dendrimer combines high solubility, well-defined molecular composition, and efficient triplet harvesting, and suffers much less from ACQ, enabling its use at much higher doping concentrations. The bar-coated LECs showed green emission with a luminance of up to 350 cd m^–2^ and an *EQE*_max_ of 1.2%, providing one of the first demonstrations of electroluminescent devices fabricated using a scalable, printing-compatible fabrication technique. In a separate collaborative work, we paired a thick inorganic CsPbCl_3_ perovskite hole-transport layer with a neat **tBuCz2m2pTRZ** emissive layer,^42^ addressing a key bottleneck in dendrimer OLEDs, namely spin-coating the EML without dissolving or disrupting the underlying organic layers. This hybrid device containing a spin-coated, host-free EML showed green electroluminescence and demonstrated that the perovskite HTL remains stable against common processing solvents. This approach presents a viable path towards multilayer solution-processed dendrimer OLED stacks.

The functional scope of TADF dendrimers extends beyond electroluminescent devices, as reported in a study by our group on dual-emissive TADF dendrimers as optical thermometers.^43^ By exploiting controlled aggregation and speciation in apolar media, dendrimers exhibited temperature- and concentration-dependent dual emission (Figure 5), enabling a remarkably wide color response from blue (monomeric emission) to yellow (aggregate emission). Indeed, the dendrimer **2GCzBPPZ**, functions as a temperature sensor over an exceptionally broad range from -70 to 70 °C in both hexane and paraffin, while maintaining TADF behavior. Collectively, these studies from our group illustrate that TADF dendrimers are not only effective emitters for SP-OLEDs but also represent a highly adaptable material platform for multifunctional optoelectronic and sensing applications, reinforcing their broader relevance within organic electronics.

**Figure 5:**
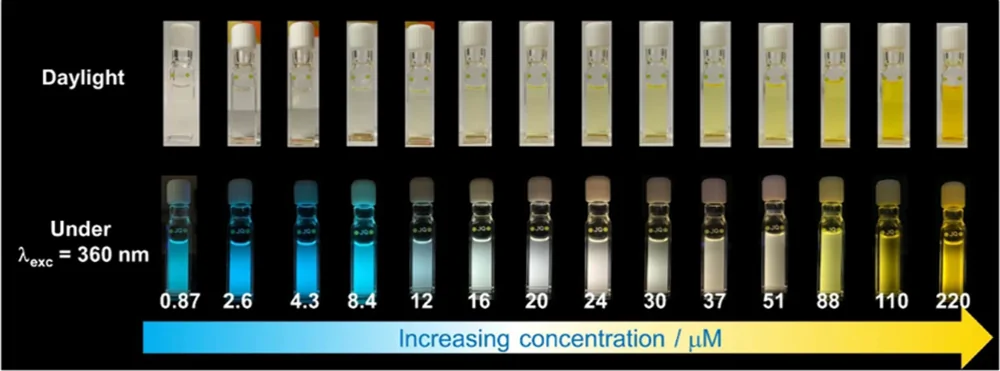
Photos of concentration-dependent fluorescence under ambient light and using a UV torch (λ_exc_ = 360 nm) of **2GCzBPPZ** in *n*-hexane. Reproduced from Ref.^43^ Available under a CC-BY 4.0 license. Copyright 2024 C. Si *et al*.

## MR-TADF Dendrimers

Interestingly, compared to diversity in molecular structure of small molecule TADF emitters used in SP-OLEDs, TADF dendrimers have been overwhelmingly based on donor–acceptor designs, and only a limited number of examples have incorporated MR-TADF cores into dendritic frameworks. This is somewhat counterintuitive, as the rigid and planar MR-TADF cores can benefit even more from dendron decoration as a means to improve solubility and mitigate ACQ.

One of the earliest systematic studies on MR-TADF dendrimers was reported by Liu *et al.*,^44^ who investigated the impact of donor dendron substitution about a boron–oxygen-nitrogen (BON) MR-TADF core by comparing the parent emitter (**BON-D0**, Figure 6) as exemplified by **BON-D1** and **BON-D2** (Figure 6). In toluene, the characteristic MR-TADF emission of the parent is preserved in both dendrimers, while there is a moderate red shift of the λ_PL_ as a result of this functionalization from 450 nm for **BON-D0** (FWHM = 23 nm) to 476 and 472 nm for **BON-D1** and **BON-D2** (FWHM = 28 and 31 nm), respectively. In 5 wt % doped films in mCP, Φ_PL_ values increase markedly upon donor dendron incorporation from 85% (**BON**-**D0**) to 98% (**BON-D2**), indicating effective suppression of non-radiative decay pathways. Time-resolved photoluminescence measurements showed τ_d_ decreasing from 157 μs (**BON-D0**) to 107 μs (**BON-D2**), which are consistent with the decrease in Δ*E*_ST_ from 0.18 to 0.10 eV (**BON-D0** to **BON-D2**). When introduced in SP-OLEDs there was a progressive increase in the *EQE*_max_ with increasing donor dendron generation from 9.7 over 13.4 to 14.9% for the devices with **BON-D0**, **BON-D1**, and **BON-D2**. However, this increase in device performance comes at the costs of color purity, the electroluminescence spectrum of the device with **BON-D2** is significantly broadened (FWHM = 61 nm) compared to those of **BON-D0** and **BON-D1** (FWHM = 39 nm), leading to a pronounced red-shift in the CIE coordinates from (0.14, 0.12) (**BON-D0**) to (0.15, 0.45) (**BON-D2**). This observation highlights an important lesson in MR-TADF dendrimer design: while donor dendron substitution improves Φ_PL_ and processability, careful control of donor dendron generation is required to avoid unintended broadening of the electroluminescent spectrum.

**Figure 6:**
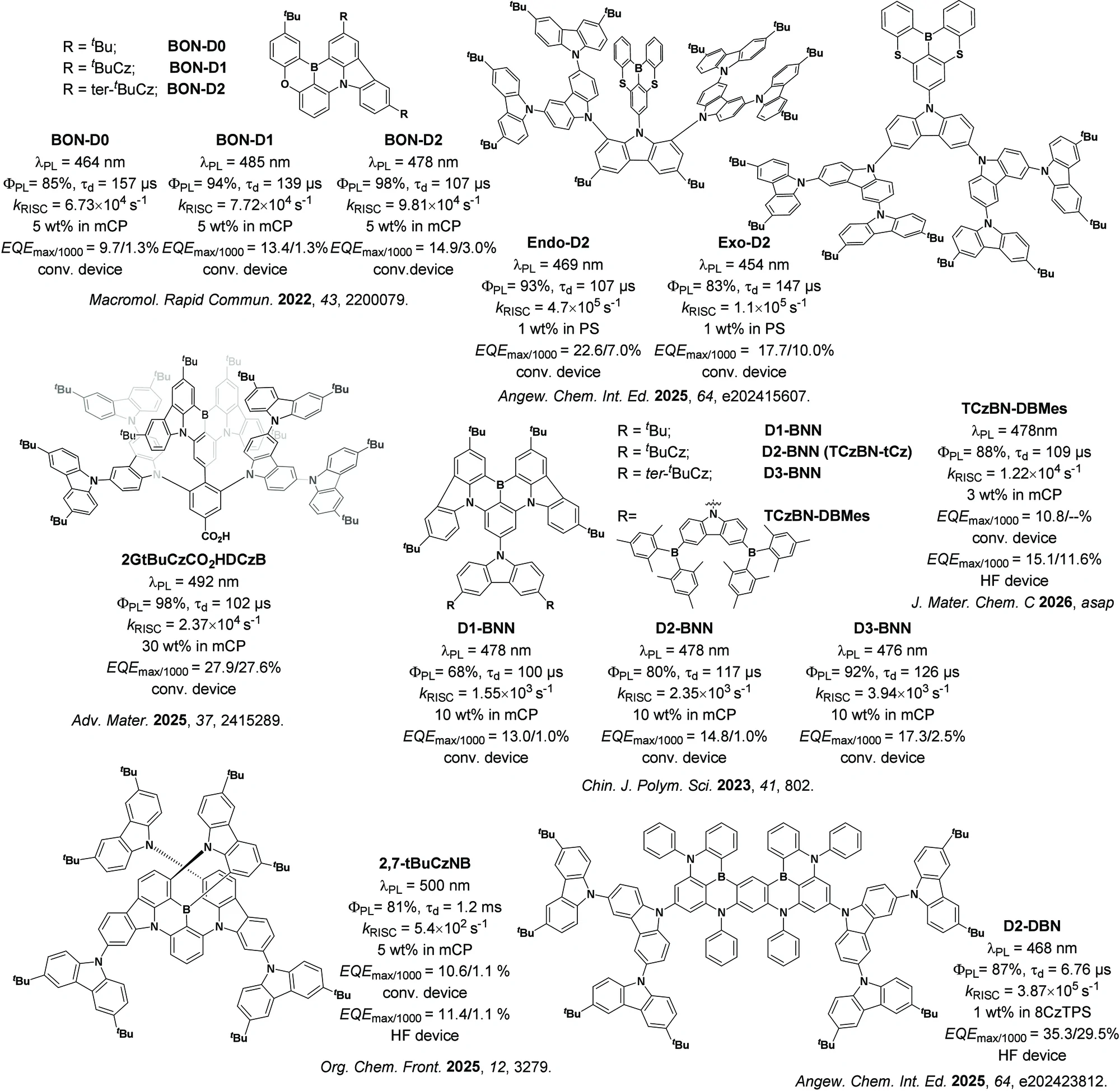
Chemical structures, key photophysical and device data of MR TADF dendrimers for SP-OLEDs.^44–50^

In a subsequent study based on dendrimer derivatives of **DtBuCzB** (Figure 1), analogues incorporating donor dendrons of increasing dendron generation (**D1-BNN**, **D2-BNN**, and **D3-BNN**, Figure 6)^45^ revealed another structure-property correlation. In contrast to prior study, the same group installed the carbazole donor dendrons at the *para* position to the boron atom on the central phenyl ring, instead of being attached to the carbazole moiety of the MR-TADF core. In 10 wt % doped films in mCP, all three emitters show narrowband blue emission, with λ_PL_ of 478 nm (**D1-BNN** and **D2-BNN**) and 476 nm (**D3-BNN**) (FWHM of 26, 24, and 24 nm, respectively). Upon increasing the doping concentration from 10 wt % to 40 wt %, the emission of **D1-BNN** and **D2-BNN** are broadened (FWHM of up to 34 nm), whereas that of the **D3-BNN** films maintains a FWHM of 24 nm across this doping concentration range, demonstrating successful suppression of aggregation. Photoluminescence quantum yields increase with increasing dendron size, from 68% for **D1**-**BNN** over 80% for **D2-BNN** reaching 92% for **D3-BNN**. Despite the trend to longer τ_d_ from 100 μs (**D1-BNN**) to 126 μs (**D3-BNN**), the corresponding *k*_RISC_ are accelerated from 1.55 to 3.94 × 10^3^ s^–1^ for **D1-BNN** to **D3-BNN**, the result of increased delayed fluorescence contribution to the overall emission. These trends indicate that increasing donor dendron generation improves excited-state kinetics not through direct modification of the MR-TADF electronic structure, but by providing enhanced steric shielding, which suppresses ACQ. In SP-OLEDs, these effects translate directly into improved performance, **D1-BNN** based devices reached an *EQE*_max_ of 13.0% at a λ_EL_ of 483 nm (FWHM of 38 nm) [CIE coordinates of (0.11, 0.36)], whereas the *EQE*_max_ progressively increases to 16.2% (**D3-BNN**) and the EL blue-shifts and narrows [λ_EL_ = 477 nm, FWHM = 24 nm, CIE coordinates of (0.11, 0.19)] for the device with **D3-BNN**). Together, these results evidence how donor dendron generation in MR-TADF dendrimers is a critical parameter for controlling aggregation, emission line width, excited-state kinetics, and ultimately device performance.

Building on the concept of MR-TADF dendrimers, the same group later introduced an *endo*-encapsulated dendrimer, **Endo-D2** (Figure 6),^47^ in which the MR-TADF core, **BSS**, is spatially shielded with carbazole donor dendrons. This design effectively shields the emissive core from intermolecular contact, almost completely suppressing aggregation-induced spectral broadening (Figure 7a). In addition, through-space interactions between the donor dendrons and the MR-TADF core were shown to reduce the Δ*E*_ST_ and accelerate RISC by approximately a factor of four compared to the *exo*-tethered analogue, with *k*_RISC_ reaching 4.7 × 10^5^ s^–1^ for **Endo-D2** (1.1 × 10^5^ s^–1^ for **Exo-D2**). As a result, SP-OLEDs based on this *endo*-encapsulated MR-TADF dendrimer achieved narrowband blue emission at λ_EL_ of 472 nm and FWHM of 38 nm [CIE coordinates of (0.13, 0.22)] accompanied by an *EQE*_max_ of 22.6%, distinguishing these devices to those with the *exo*-analog, with a λ_EL_ of 460 nm and FWHM of 41 nm [CIE coordinates of (0.15, 0.17)] and an *EQE*_max_ of 17.7%. These results show that by encapsulation of the MR-TADF core, aggregation and therefore emission quenching processes within the device can be mitigated, but at the same time comes at the cost of red-shifted emission.

**Figure 7:**
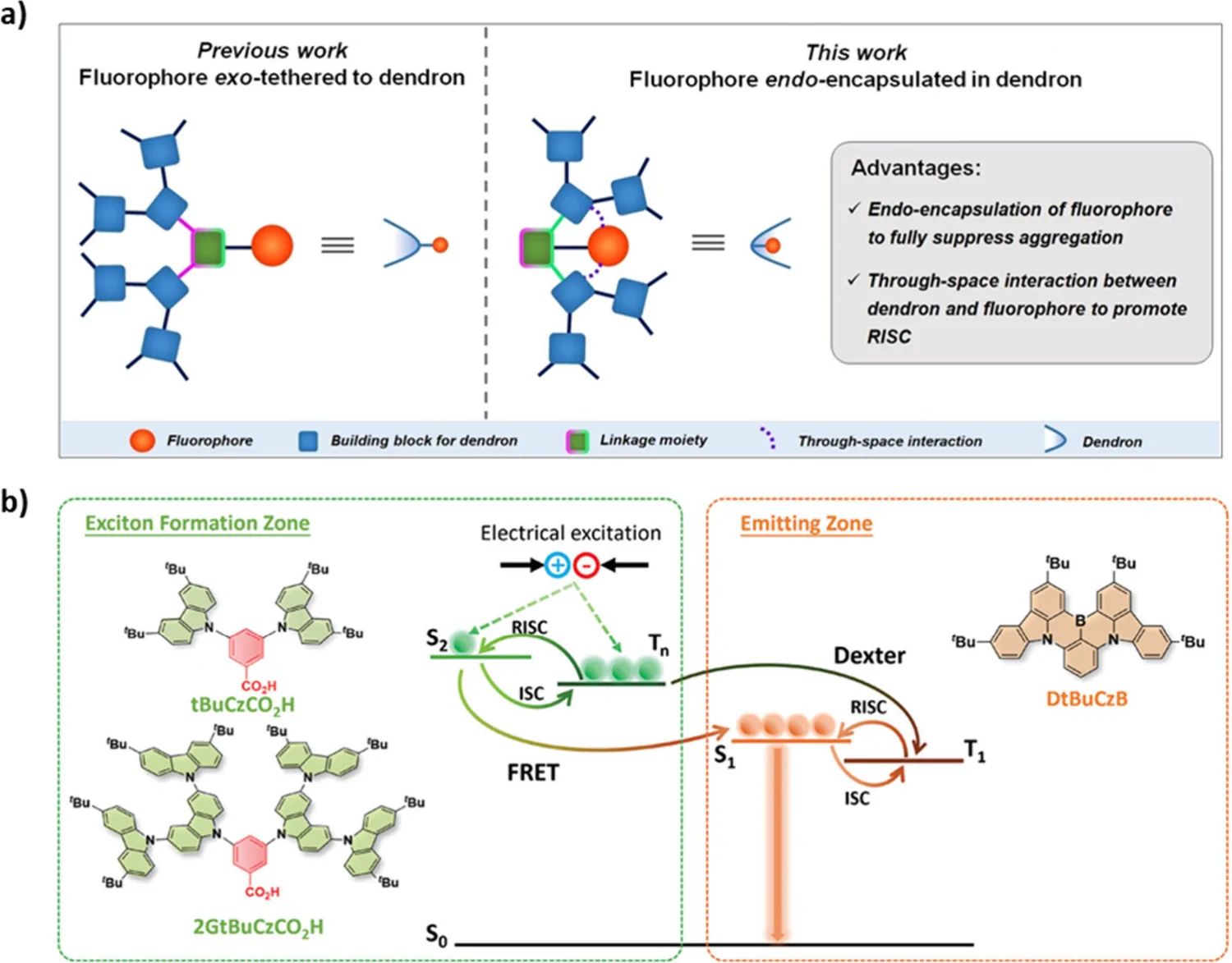
Molecular design for endo-encapsulated multi-resonance (MR) dendrimers. Adapted with permission from Ref.^47^ Copyright 2025 John Wiley and Sons. b) The design concept of the intermolecular energy transfer strategy between the peripheral pendant, **tBuCzCO**_**2**_ **H** and **2GBuCzCO**_**2**_ **H**, as well as the central MR-TADF core **DtBuCzB**. Adapted from Ref.^46^ Available under a CC-BY 4.0 license. Copyright 2025 S. Wu *et al*.

Adopting a related but conceptually distinct strategy, our group translated intramolecular exciton-management concepts previously established for small-molecule MR-TADF emitters^26,29^ into a dendritic framework. In these MR-TADF dendrimers, the **DtBuCzB** (Figure 1) core is covalently coupled to carbazole- dendron-substituted benzoate donor–acceptor units that act as fast-RISC antennae. Importantly, in **2GtBuCzCO**_**2**_ **HDCzB** ^46^ (Figure 6) the photophysical properties of the **DtBuCzB** core are preserved, evidenced by narrowband emission in solution with λ_PL_ of 485 nm and a FWHM of 22 nm, comparable to those of **DtBuCzB** (λ_PL_ = 483 nm, FWHM = 23 nm). In doped films (30 wt % in mCP), **2GtBuCzCO**_**2**_ **HDCzB** has a near unity Φ_PL_ of 98%, while maintaining narrowband emission (FWHM = 27 nm), indicating effective suppression of ACQ, even at these relatively high doping concentrations for MR-TADF emitters. Time-resolved PL measurements revealed a τ_d_ of 102 μs for **2GtBuCzCO**_**2**_ **HDCzB**. The corresponding *k*_RISC_ of 2.37 × 10^4^ s^–1^ indicates efficient upconversion enabled by this intramolecular FRET design. These values reflect efficient triplet harvesting on the peripheral donor–acceptor units followed by quantitative intramolecular Förster resonance energy transfer to the **DtBuCzB** core, from which the narrowband emission originates (Figure 7b). Beyond the improved exciton kinetics, the dendritic architecture provides an additional level of morphological control as **2GtBuCzCO**_**2**_ **HDCzB** retains a narrowband emission even at doping concentrations as high as 70 wt %, a regime typically inaccessible for small-molecule MR-TADF emitters. These combined effects translate directly into high-performance SP-OLEDs. Devices employing 30 wt % **2GtBuCzCO**_**2**_ **HDCzB** in mCP exhibited green λ_EL_ of 495 nm and a FWHM of 27 nm, corresponding to CIE coordinates of (0.08, 0.53). These devices showed an *EQE*_max_ of 27.9%, with remarkably low efficiency roll-off, achieving an *EQE*_2000_ of 27.6%. Notably, host-free SP devices with **2GtBuCzCO**_**2**_ **HDCzB** achieved an *EQE*_max_ of 24.0% and maintained an *EQE*_1000_ of 20.2%, underscoring the effectiveness of combining intramolecular FRET with dendritic encapsulation. Overall, this work demonstrates that intramolecular exciton-management strategies simultaneously address excited-state kinetics and solid-state aggregation, enabling SP-OLED performances that approach those of state-of-the-art VD devices.

A landmark demonstration of the potential of MR-TADF dendrimers for high-performance SP-OLEDs was reported by Zhang *et al*. in 2025.^49^ In their work, the authors combined a dendritic MR-TADF emitter **D2-DBN** (Figure 6) with a dendritic silicon-based host **8CzTPS** to address two central limitations of SP MR-TADF devices: ACQ and poorly controlled TDM orientation. Through electrostatic interactions between host and emitter, a highly preferential horizontal TDM orientation was induced in spin-coated films, yielding a remarkable horizontal dipole ratio of 83%, comparable to values typically achieved in VD films. Simultaneously, dendron encapsulation effectively suppressed aggregation, resulting in a Φ_PL_ of 99% for the emissive layer. When employed as the terminal emitter in HF SP-OLEDs, using the fast-RISC assistant dopant **5** *t* **BuCzTRZ** to compensate for the intrinsically slow RISC of **D2-DBN**, the devices achieved an exceptionally high *EQE*_max_ of 35.3% and low efficiency roll-off (*EQE*_1000_ of 29.5%), together with narrowband blue emission at λ_EL_ of 471 nm (FWHM of 17 nm), corresponding to CIE coordinates of (0.14, 0.18). These results exemplify that MR-TADF dendrimers, when combined with a suitably interacting host, can deliver SP HF OLEDs performing on par with VD devices.

So far, all of the highlighted MR-TADF dendrimers have relied on 9,3′:6′,9″-*ter*carbazole-based donor dendrons. However, alternative dendron designs can offer additional advantages. Recently, we introduced a novel MR-TADF dendrimer based on 3,3″,6,6″-tetra-*tert*-butyl-9′H-9,2′:7′,9″-*ter*carbazole, **2,7-tBuCzNB** (Figure 6),^48^ where a modification of the regiochemistry within the donor dendron introduces greater steric hindrance and more pronounced helicity. This structural change effectively suppresses aggregation-caused quenching and delivers SP-OLEDs having *EQE*_max_ of 10.6%, where the emitter doping concentration can be as high as 10 wt %.

In a separate study, we explored the decoration of peripheral carbazole groups in a 9,3′:6′,9″-*ter*carbazole with dimesitylborane accepting groups to form **TCzBN-DBMes** (Figure 6).^50^ Notably, this modification preserved the photophysical properties (λ_PL_ = 478 nm, Φ_PL_ = 88%, *k*_RISC_ = 1.22 × 10^4^ s^–1^) and device performance (λ_EL_ = 477 nm, *EQE*_max/500_ = 10.8/1.5%) of the reference dendrimer **TCzBN-tCz** (also known as **D2-BNN**, λ_EL_ = 478 nm, *EQE*_max/500_ = 11.7/2.0%), while producing a narrower EL spectrum (FWHM of 24 nm; CIE coordinates of (0.12, 0.20)] than the corresponding carbazole-only analogue [FWHM of 27 nm; CIE coordinates of (0.12, 0.23)]. These results highlight that acceptor-functionalized dendrons provide an additional design handle to finetune the electroluminescence behavior without compromising the intrinsic MR-TADF emission or device efficiency.

These studies (summarized in Table **S3**) evidence that MR-TADF dendrimers have evolved from early proof-of-concept systems into a highly effective materials platform for SP-OLEDs. While initial work relied on straightforward dendron attachment to improve solubility and suppress aggregation, more recent strategies deliberately exploit dendritic architectures to control solid-state organization, exciton utilization, and transition dipole orientation. *Endo*-encapsulation, intramolecular FRET-assisted exciton harvesting, and rational host-emitter pair design have collectively enabled substantial improvements in Φ_PL_, RISC kinetics, mitigated ACQ, and device efficiency roll-off. Notably, the combination of MR-TADF dendrimers acting as terminal emitters with assistant dopants within HF devices has yielded SP-OLEDs with performances approaching those of state-of-the-art VD devices. At the same time, ongoing exploration of alternative dendron architectures, including sterically demanding and acceptor-functionalized dendrons, highlights the versatility of dendrimer design in addressing aggregation and spectral control. These advances firmly establish MR-TADF dendrimers as a key material class for overcoming the intrinsic limitations of small-molecule MR-TADF emitters in SP-OLEDs.

## Conclusions and Outlook

In this account, we have provided an overview of the recent developments in the field of SP TADF materials, with a main focus on MR-TADF emitters and TADF dendrimers, which when combined together, in our view, represent the most promising emitter design concept for high-performance SP-OLEDs. Looking back, the field has made progress along three fronts: 1. Developed strategies that accelerate RISC in MR-TADF emitters; 2. Produced approaches that improve emitter orientation in SP layers; and 3. Evolved dendrimer design pattern that suppress ACQ and retain solution processability. Beyond ACQ, small molecule SP emitters remain intrinsically prone to phase separation and crystallization within the host matrix during device operation, both of which introduce device degradation pathways.^51^ Host-guest engineering that minimizes intermolecular interactions, thermally or photochemically cross-linkable substituents, and the macrocyclic/dendritic encapsulation strategies discussed above have each been shown to suppress crystallization and improve the morphological robustness of solution-processed films.^12,51,52^ These issues can each already be overcome to a certain extent; however, orientation control remains as major challenge in SP layers. We and others have revealed that like their VD counterparts, the performance of SP-OLEDs can benefit from HF strategies. The examples to date demonstrate that a smart molecular design can play a huge role in closing the performance gap between VD and SP devices. Looking ahead, we foresee the roadmap for the field unfolding in three stages. Firstly, one priority should lie in merging MR-TADF cores with FRET and orientation-control motifs within a singular dendrimer framework. Secondly, there will be an effort to expand the existing emission color gamut to meet the industry-required color points for red, green and blue. Thirdly, integration of said emitters into fully SP-OLEDs, alongside compatible host and charge transport materials. This materials design progress must be matched by parallel advances in device engineering that are distinct from VD-OLEDs. Because solution-deposited layers can redissolve underlying films, multilayer SP-OLED architectures generally require orthogonal solvent processing or thermally/photochemically cross-linkable interlayers to fabricate functional stacks.^53^ Operational stability for such fully solution-processable devices remains comparatively underexplored. Scalable, non-vacuum manufacturing routes, including slot-die and inkjet printing, are increasingly compatible with roll-to-roll production and are expected to be decisive to deliver on the cost and throughput advantages that motivate the development of SP-OLEDs in the first place.^54,55^ We believe that the nascent field of MR TADF dendrimers will remain one of key emitter design paradigms for SP TADF emitters to guide this progress. SP-OLEDs are on the cusp of commercialization,^56^ and with this advent will come a demand for ever increasing demand for high-performance materials and TADF dendrimers should have a place at the table, given their illustrated promise to date.

## Supplementary Material



## Notes

Performance of solution-processed OLEDs using small molecule TADF, MR-TADF dendrimers, and TADF dendrimers as emitters (PDF)
