# Supplementary material for: Closing the Gap: Multiresonant TADF Emitters and TADF Dendrimers in Solution-Processed OLEDs
Source: Acc Mater Res. 2026 Sep 25;7(10):1103–16. doi: 10.1021/accountsmr.6c00190 (PMC13711631; doi:10.1021/accountsmr.6c00190)
Supplement: Supplementary file 1 [file mr6c00190_si_001.pdf]

Closing the Gap: Multiresonant TADF Emitters and TADF Dendrimers in Solution-Processed OLEDs

Mahni Fatahi[a] *and Eli Zysman-Colman**[a]

[a]Organic Semiconductor Centre, EaStCHEM School of Chemistry, University of St Andrews, St Andrews, UK, KY16 9ST. E-mail: eli.zysman-colman@st-andrews.ac.uk

Table **S1**. Performance of solution-processed OLEDs using small molecule TADF as emitters

Emitter	λ_{PL} / nm	Φ_{PL} / %	τ_d / µs	k_{RISC} / s^{-1}	Device type	λ_{EL} / nm	EQE_{max} / %	EQE_{1000} / %	Ref.
**DOBN**	449	98	21.7	7.5×10^4	conv.<br>HF	449<br>447	6.8<br>27.7	--<br>--	1
**H-FOBN**	476	82	75.6	6.4×10^4	conv.	460	9.2	--	2
**Me-FOBN**	464	86	67.2	7.5×10^4	conv.	458	11.3	--	2
**DtBuCzB**	481	91	68.6	n/a	conv.*	488	21.6*	5.3*	3
**tButHCzB**	491	61	95.0×10^3	1.44×10	--	--	--	--	4
**DtButHCzB**	490	57	15.4×10^4	2.95×10	--	--	--	--	4
**DiKTa-LC**	512	52	156	n/a	conv.	492	13.6	--	5
**DICz-DMOC**	457	51	3.14×10^3	0.3×10^3	conv.	468	10.0	4.9	6
**DICz-DOD**	457	28	3.61×10^3	0.2×10^3	conv.	471	5.2	2.6	6
***m*-DT-BCzBN**	496	99	18.2	1.4×10^5	conv.	492	27.8	19.8	7
***p*-DT-BCzBN**	497	98	20.5	1.0×10^5	conv.	494	25.4	4.5	7
**TRZ-SpAc-DBN**	499	89	12.6	1.30×10^5	conv.<br>HF	495<br>492	6.4<br>20.7	1.9<br>20.2	8
**DOBNA-SpAc-DBN**	500	96	13.9	1.16×10^5	conv.<br>HF	495<br>492	6.8<br>11.7	1.4<br>11.7	8
**PCP-*t*CzBN**	493	77	53.5	1.73×10^4	conv.	489	15.5	2.6	9

***po*-PCP-*t*CzBN-DiKTa**	495	61	29.3	5.31×10^{4}	conv.	494	23.0	9.0	9

*vacuum-deposited device

Table **S2**. Performance of solution-processed OLEDs using TADF dendrimers as emitters.

Emitter	λ_{PL} / nm	Φ_{PL} / %	τ_d / µs	k_{RISC} / s^{-1}	Device type	λ_{EL} / nm	EQE_{max} / %	EQE_{1000} / %	Ref.
**G2TAZ**	500	52	6.2	n/a	conv.	510	2.4	1.9	10
**G3TAZ**	500	31	3.4	n/a	conv.	508	3.4	1.9	10
**G4TAZ**	495	8.5	4.3	n/a	conv.	504	1.5	--	10
**tBuCz2mTRZ**	516	59	1.1	5.4×10^{5}	conv.	516	19.9	--	11
**tBuCz3mTRZ**	536	62	2.2	6.5×10^{5}	conv.	536	23.7	15.0	11
**tBuCz4mTRZ**	536	67	1.7	4.3×10^{5}	conv.	536	23.8	15.0	11
**2FBP2GCz**	560	68	3.2×10^{3}	0.04×10^{4}	conv.	545	3.1	2.6	12
					HF	540	18.7	17.3	
**2FBP2DPACz**	615	44	51	2.13×10^{4}	conv.	585	4.0	2.3	12
					HF	580	17.0	16.8	
**2GCzBP**	570	77	0.3×10^{3}	0.36×10^{4}	conv.	561	2.7	2.3	12
					HF	555	13.5	12.3	
**2DPACzBP**	640	28	1.3	79.8×10^{4}	conv.	640	7.8	7.5	12
					HF	605	20.0	11.8	
**DOBNA-SpAc-DCz**	474	93	2.0	1.71×10^{6}	conv.	474	29.4	--	13
**tBuCz2pTRZ**	481	61	1.2	8.3×10^{5}	conv.	500	18.5	7.5	14
**tBuCz2mTRZ**	483	59	1.1	9.1×10^{5}	conv.	516	19.9	--	14
**tBuCz2m2pTRZ**	520	86	1.1	9.1×10^{5}	conv.	540	28.7	12.5	14

Table **S3**. Performance of solution-processed OLEDs using MR-TADF dendrimers as emitters.

Emitter	λ_{PL} / nm	Φ_{PL} / %	τ_d / μs	k_{RISC} / s^{-1}	Device type	λ_{EL} / nm	EQE_{max} / %	EQE_{1000} / %	Ref.
**BON-D0**	464	85	157	6.73×10^4	conv.	460	9.7	1.3	15
**BON-D1**	485	94	139	7.72×10^4	conv.	488	13.4	1.3	15
**BON-D2**	478	98	107	9.81×10^4	conv.	487	14.9	3.0	15
**Endo-D2**	469	93	107	4.7×10^5	conv.	472	22.6	7.0	16
**Exo-D2**	454	83	147	1.1×10^5	conv.	460	17.7	10.0	16
**2GtBuCzCO$_2$HDCzB**	492	98	102	2.37×10^4	conv.	495	27.9	27.6	17
**D1-BNN**	478	68	100	1.55×10^3	conv.	483	13.0	1.0	18
**D2-BNN**	478	80	117	2.35×10^3	conv.	479	14.8	1.0	18
**D3-BNN**	476	92	126	3.94×10^3	conv.	477	17.3	2.5	18
**TCzBN-DBMes**	478	88	109	1.22×10^4	conv.	477	10.8	--	19
					HF	476	15.1	11.6	
**2,7-tBuCzNB**	500	81	1.2×10^3	5.4×10^2	conv.	503	10.6	1.1	20
					HF	500	11.4	1.1	
**D2-DBN**	469	87	6.76	3.87×10^5	HF	471	35.3	29.5	21

References

(1) Jin, J.; Chen, M.; Jiang, H.; Zhang, B.; Xie, Z.; Wong, W.-Y. Construction of Hybrid Long- and Short-Range Charge Transfer Excitations for High-Performance Deep-Blue Electroluminescence with CIEy < 0.04 and EQE > 35%. *ACS Materials Letters* **2024**, *6* (8), 3246-3253.
(2) Song, X.; Shen, S.; He, B.; Zou, S.; Vaitusionak, A.A.; Kostjuk, S.V.; Wang, Y.; Wang, Y.; Zhang, Y. Dual Multiresonance Core Strategy Enable Efficient Pure Blue Organic Light-Emitting Devices Based on Fluorene Linkages. *Adv. Optical Mater.* **2024**, *12* (32), 2401505.
(3) Xu, Y.; Cheng, Z.; Li, Z.; Liang, B.; Wang, J.; Wei, J.; Zhang, Z.; Wang, Y. Molecular-Structure and Device-Configuration Optimizations toward Highly Efficient Green Electroluminescence with Narrowband Emission and High Color Purity. *Adv. Optical Mater.* **2020**, *8* (9).

(4) Fatahi, M.; Mckay, A.P.; Cordes, D.B.; Zysman-Colman, E. Saturated carbazole-embedded BN-aromatic systems as narrowband sky-blue emitters. *J. Mater. Chem. C* **2025**, *13* (40), 20549-20558.
(5) Chen, D.; Tenopala-Carmona, F.; Knoller, J.A.; Mischok, A.; Hall, D.; Madayanad Suresh, S.; Matulaitis, T.; Olivier, Y.; Nacke, P.; Giesselmann, F.; Laschat, S.; Gather, M.C.; Zysman-Colman, E. Mesogenic Groups Control the Emitter Orientation in Multi-Resonance TADF Emitter Films. *Angew. Chem. Int. Ed.* **2023**, *62* (16), e202218911.
(6) De, J.; Gupta, A.K.; Litvinas, D.; Tenopala-Carmona, F.; Lehmann, M.; Gather, M.C.; Zysman-Colman, E. Harnessing the Orientation of Columnar Discotic Liquid Crystals for Narrowband Blue Emission with Enhanced Out-Coupling Efficiency Toward Improvement of SP-OLEDs Performance. *Adv. Funct. Mater.* **2026**, *36* (36), e28081.
(7) Yang, Z.; Li, S.; Hua, L.; Ying, S.; Liu, Y.; Ren, Z.; Yan, S. Intramolecular charge transfer assisted multi-resonance thermally activated delayed fluorescence emitters for high-performance solution-processed narrowband OLEDs. *Chem. Sci.* **2025**, *16* (9), 3904-3915.
(8) Fatahi, M.; Barman, D.; Tsuchiya, Y.; Adachi, C.; Zysman-Colman, E. Intramolecular FRET Cascades Enable Small Efficiency Roll-Off in Solution-Processed OLEDs. *Adv. Optical Mater.* **2026**, *14* (19), e71254.
(9) Xu, Y.; Chen, D.; Wu, S.; Black, D.J.; Wang, H.; Pal, R.; Zhang, X.; Zysman-Colman, E. Accelerating Reverse Intersystem Crossing by Bridging Two Multiresonant Thermally Activated Delayed Fluorescence Emitters with [2.2]Paracyclophane. *CCS Chemistry* **2025**, *7* (12), 3635-3649.
(10) Albrecht, K.; Matsuoka, K.; Fujita, K.; Yamamoto, K. Carbazole dendrimers as solution-processable thermally activated delayed-fluorescence materials. *Angew. Chem. Int. Ed.* **2015**, *54* (19), 5677-5682.
(11) Sun, D.; Saxena, R.; Fan, X.; Athanasopoulos, S.; Duda, E.; Zhang, M.; Bagnich, S.; Zhang, X.; Zysman-Colman, E.; Kohler, A. Regiochemistry of Donor Dendrons Controls the Performance of Thermally Activated Delayed Fluorescence Dendrimer Emitters for High Efficiency Solution-Processed Organic Light-Emitting Diodes. *Adv. Sci.* **2022**, *9* (20), e2201470.
(12) Si, C.; Sun, D.; Matulaitis, T.; Cordes, D.B.; Slawin, A.M.Z.; Zysman-Colman, E. Rational molecular design of efficient yellow-red dendrimer TADF for solution-processed OLEDs: a combined effect of substitution position and strength of the donors. *Sci. China Chem.* **2024**, *67* (5), 1613-1623.
(13) Fatahi, M.; Yasuda, Y.; Kondo, R.; Kaji, H.; Zysman-Colman, E. Donor Extended Blue TADF Dendrimer for High-Performance Solution-Processed OLEDs. *Adv. Sci.* **2026**, *n/a* (n/a), e76130.
(14) Sun, D.; Duda, E.; Fan, X.; Saxena, R.; Zhang, M.; Bagnich, S.; Zhang, X.; Kohler, A.; Zysman-Colman, E. Thermally Activated Delayed Fluorescent Dendrimers that Underpin High-Efficiency Host-Free Solution-Processed Organic Light-Emitting Diodes. *Adv. Mater.* **2022**, *34* (23), e2110344.
(15) Liu, J.; Chen, L.; Wang, X.; Yang, Q.; Zhao, L.; Tong, C.; Wang, S.; Shao, S.; Wang, L. Multiple Resonance Dendrimers Containing Boron, Oxygen, Nitrogen-Doped Polycyclic Aromatic Emitters for Narrowband Blue-Emitting Solution-Processed OLEDs. *Macromol. Rapid Commun.* **2022**, *43* (16), e2200079.

(16) Chang, Y.; Zhang, K.; Zhao, L.; Wang, X.; Wang, S.; Shao, S.; Wang, L. Endo-Encapsulated Multi-Resonance Dendrimers with Through-Space Interactions for Efficient Narrowband Blue-Emitting Solution-Processed OLEDs. *Angew. Chem. Int. Ed.* **2025**, *64* (3), e202415607.
(17) Wu, S.; Chen, D.; Zhang, X.H.; Sun, D.; Zysman-Colman, E. A Multiresonant Thermally Activated Delayed Fluorescent Dendrimer with Intramolecular Energy Transfer: Application for Efficient Host-Free Green Solution-Processed Organic Light-emitting Diodes. *Adv. Mater.* **2025**, *37* (8), e2415289.
(18) Liu, J.-P.; Chen, L.; Zhao, L.; Tong, C.-Y.; Wang, S.-M.; Shao, S.-Y.; Wang, L.-X. Carbazole-based Multiple Resonance Dendrimers with Narrowband Blue Emission for Solution-Processed OLEDs. *Chin. J. Polym. Sci.* **2023**, *41* (5), 802-810.
(19) Fatahi, M.; Bareikaite, K.; Choudhary, P.; Hu, Y.; Danos, A.; Tsuchiya, Y.; Adachi, C.; Monkman, A.P.; Zysman-Colman, E. Narrowband sky-blue emission from acceptor-extended MR-TADF dendrimers. *J. Mater. Chem. C* **2026**.
(20) Wang, J.; Yasuda, Y.; Ren, Y.; Kondo, R.; Cordes, D.B.; Kaji, H.; Zysman-Colman, E. A multi-resonant thermally activated delayed fluorescence emitter with a twisted second-generation carbazole dendron showing suppressed concentration quenching and its use in solution-processed organic light-emitting diodes. *Org. Chem. Front.* **2025**, *12* (10), 3279-3287.
(21) Zhang, K.; Wang, X.; Wang, M.; Wang, S.; Wang, L. Solution-Processed Blue Narrowband OLED Devices with External Quantum Efficiency Beyond 35 % through Horizontal Dipole Orientation Induced by Electrostatic Interaction. *Angew. Chem. Int. Ed.* **2025**, *64* (13), e202423812.
